# Timely delivery of laboratory efficiency information, Part II: Assessing the impact of a turn-around time dashboard at a high-volume laboratory

**DOI:** 10.4102/ajlm.v9i2.948

**Published:** 2020-04-23

**Authors:** Naseem Cassim, Lindi M. Coetzee, Manfred E.E. Tepper, Louella Perelson, Deborah K. Glencross

**Affiliations:** 1 National Health Laboratory Service (NHLS), Johannesburg, South Africa; 2 Department of Molecular Medicine and Haematology, Faculty of Health Sciences, University of the Witwatersrand, Johannesburg, South Africa

**Keywords:** turn-around-time, laboratory efficiency, pathology, laboratory medicine

## Abstract

**Background:**

In South Africa’s National Health Laboratory Service, ad hoc mean turn-around time (TAT) reporting is an important indicator of performance. However, historic static TAT reporting did not assess very long or very short times. An interactive TAT dashboard was developed using the following TAT measures; (1) median, (2) 75th percentile and (3) percentage of within cut-off TAT to allow for improved differentiation of TAT performance.

**Objectives:**

The objective of our study was to demonstrate increased efficiency achieved by using an interactive TAT dashboard.

**Methods:**

A retrospective descriptive study design was used. Creatinine TAT outcomes were reported over 122 weeks from a high-volume laboratory in Gauteng, South Africa. The percentage of within cut-off and 75th percentile TAT were analysed and reported using Microsoft Excel. A focus group session was used to populate a cause and effect diagram.

**Results:**

The percentage of within cut-off TAT increased from 10% in week 4 to 90% and higher from week 81. The 75th percentile decreased from 10 hours in week 4 to under 5 h from week 71. Component TAT analysis revealed that the 75th percentile testing was 5 h or longer for weeks 4, 5 and 48. The 75th percentile review TAT ranged from 1 h to 15 h. From week 41, the review TAT was under 1 h.

**Conclusion:**

Our study demonstrated that the use of an interactive TAT dashboard coupled with good management can dramatically improve TAT and efficiency in a high-volume laboratory.

## Introduction

Turn-around time (TAT) is an important performance indicator of laboratory efficiency to deliver patient results.^1^ In the South African National Health Laboratory Services, ad hoc mean TAT reports were previously produced for laboratory managers. These TAT reports assessed performance based on the National Health Laboratory Service global annual performance plan (APP) TAT cut-offs specific for individual tests.^2^ Reports were provided intermittently in a static form that assessed central tendency only (i.e. the tail size was not reported) and did not allow for drilling functionality to access additional, more detailed, information to direct meaningful corrective action (i.e. laboratory or sample-level TAT breakdown). To improve on these TAT reporting systems, Coetzee et al. used three additional measures to assess TAT efficiency: (1) median TAT, (2) 75th percentile TAT (tail size) and (3) percentage of within cut-off TAT.^3^ These measures accurately assessed outliers as tail size and could be used by laboratories to address workflow issues and identify testing delays for intervention. Tail size refers to the volume of samples in a positively skewed data distribution that has a long tail to the right. These samples often have a much higher TAT value than the central tendency (median) for this data distribution. Tail size can be measured as the percentage of samples that exceed a defined TAT cut-off in hours or as a percentile.

Initially, the three measures described above were reported in Microsoft Excel (Redmond, Washington, United States) worksheet format from August 2016 to June 2017.^4^ Thereafter, from July 2017, an interactive dashboard was developed that reported TAT data for a basket of tests using the Microstrategy Desktop (Tysons, Virginia, United States) analytics tool.^5^ Previously, the static reports and, more recently, the interactive dashboard reports are distributed to area (province), business (district) and laboratory managers. Data can be reviewed in the interactive dashboard reports across the provincial, district or laboratory levels through drilling functionality, which makes it possible to slice through a data hierarchy to reveal additional details^6^ contained within the aggregated data. In this way, TAT data presented can be visualised at the national, provincial and laboratory level on the same dashboard page. The approach allows various levels of manager to drill down from a ‘bird’s-eye’ view of TAT performance nationally to the provincial or individual laboratory level.

Within the dashboard, TAT can be viewed for a basket of tests including routine haematology full blood count with platelet and differential testing, international normalised ratio, activated prothrombin testing and D-dimers, chemical pathology testing including urea and electrolytes, liver function testing, glucose, cholesterol, among others, as well as microbiology testing for HIV (HIV viral load, HIV DNA polymerase chain reaction), tuberculosis (Xpert MTB/RIF [mycobacterium tuberculosis DNA/resistance to rifampicin) and syphilis (rapid plasma reagin and *Treponema pallidum* antibodies) and, lastly, cluster of differentiation 4 (CD4) testing. Proxy marker analytes are used to assess performance of the respective matched assay panel, for example creatinine is used as the proxy test to review the urea and electrolytes performance. Each test has its own predetermined TAT determined at the local level according to the level of care, with absolute national APP cut-offs noted.

Global TAT outcomes for each test are reported according to specifically stipulated, organisation-determined TAT APP at the national level and are described elsewhere.^2,7^ National APP cut-offs are set bearing in mind the multi-tiered service that accommodates reporting from primary health care referral to large tertiary centres that may offer emergency services, and do not necessarily reflect the respective individual, laboratory-stipulated TAT, which may be self-determined by laboratories based on their local clinical needs.

Armed with the knowledge of TAT and which tests are identified as poor performers in the interactive dashboard, laboratory managers can identify and address areas of concern through review of the contributing causes.^8^ This is achieved through root cause analysis, a method of problem-solving used to identify the root causes (faults or problems) and determine the most probable underlying causes of error.^8^ The ultimate aim of root cause analysis in TAT monitoring is to formulate corrective actions that either mitigate or eliminate the identified causes to return TAT efficiency and performance to acceptable levels.

The aim of this study was to report on the impact of an interactive dashboard that provides weekly information about TAT and enables laboratory and senior managers to monitor TAT and identify problematic areas for corrective action. The hypothesis was that an interactive TAT dashboard delivering week-by-week information about laboratory TAT provides the impetus for continuous service review and implementation of appropriate corrective action, where required, to ensure the timeliness of laboratory reporting. Data are presented from a single, busy, routine automated clinical pathology laboratory at a large regional hospital to reveal how the described TAT dashboard served to continually highlight ongoing TAT delays for urea and electrolyte (creatinine) result reporting and, ultimately, facilitated sustained corrective action.

## Methods

### Ethical considerations

Ethics clearance was obtained from the University of the Witwatersrand (study approval number: M1706108). No patient identifiers were extracted with data.

### Study design and samples used

A retrospective descriptive study design was used to analyse laboratory data and highlight the impact of interventions by observing trends. Qualitative focus group sessions were used to unpack the root causes of poor performance. Convenience sampling was used. For the purpose of this study, the TAT performance for creatinine testing, which had poor TAT at the start of the study, was used to demonstrate how dashboard monitoring of TAT could highlight and impact the TAT. Creatinine testing outcomes were reported with an APP cut-off of 90% within 5 hours.^2^ Weekly TAT data, from the week ending 07 August 2016 (01 August 2016 to 07 August 2016) to the week ending 02 December 2018 (26 November 2018 to 02 December 2018) was reviewed (122 weeks).

### Data extraction and turnaround time definition

The data extract contained the following variables: (1) report week ending date, for example 23 October 2016 (Monday to Sunday), (2) laboratory name, (3) test method name, (4) TAT cut-off, (5) test volumes, (6) percentage of within cut-off TAT, (7) median TAT, (8) 75th percentile TAT, (9) inter-laboratory referral 75th percentile TAT, (10) testing 75th percentile TAT and (11) review 75th percentile TAT. All TAT 75th percentile values were reported in hours. Each week was numbered, that is, 1–122. TAT data refer to total TAT (i.e. time of first registration to time of result release after review) if not otherwise specified for TAT components. All data were prepared and analysed using Microsoft Excel (Redmond, Washington, United States).^4^ The testing TAT time interval was calculated from time of registration in the testing laboratory to time of result generation on the analyser interface. Review TAT (TST-TO-RVW [test-to-review]) is the time taken by a senior technologist to review the patients’ results on the laboratory information system, making sure all quality checks were adequately performed before releasing (authorising) the patients report. The recorded time interval, that is, the review TAT, was calculated afterwards for each individual sample outcome from the time of result generation to the time of authorisation or review.

### Percentage within cut-off turnaround time analysis

The percentage of within cut-off TAT was calculated as the total number of samples meeting the organisation’s TAT cut-off criteria of 5 h for urea and electrolytes testing divided by the total number of tests performed, expressed as a percentage, per week. The results were reported as a line chart (indicating the week number and APP cut-off of 90%). Data were segmented into three phases: (1) baseline: week 1 to 44 (week ending 04 June 2017), (2) dashboard intervention: week 45 to 63 (week ending 15 October 2017) and (3) post-intervention from week 64 to 122 (week ending 02 December 2018). The dashboard intervention period indicates the switch from using an Excel worksheet to the interactive dashboard.

### 75th percentile turnaround time analysis

The 75th percentile was calculated for total TAT per week, as well as for TAT components, that is, testing and review. As tests were local hospital-based and not referred from surrounding laboratories, the pre-analytical TAT component was not applicable. When samples are referred, the pre-analytical TAT measures the interval (time taken to transport the sample between laboratories) from registration at the source (the laboratory where the sample was received) to the testing laboratory. Results from this analysis were plotted as 75th percentile, per testing week, for both total and component TAT.

### Root cause analysis

The root cause analysis diagram was used to identify potential factors causing poor TAT performance.^9^ Causes were grouped into the following headings: (1) equipment and supplies, (2) environmental, (3) rules, policies or procedures and (4) staff or personnel. Focus group meetings were arranged with the laboratory manager and section supervisors to identify causes and to populate the cause and effect diagram. A voice recorder was used to create the cause and effect diagram using Microsoft Visio (Redmond, Washington, United States).^5^

### Results

This laboratory performed 326 081 tests for the financial period 2016/2017, 341 760 tests for 2017/2018 and 399 538 tests for 2018/2019. Assuming 24/7 operations, this equates to between 894 and 1095 tests per day (Booplal N 2019, personal communication). Prior to the implementation of the interactive dashboard, weekly TAT data were extracted from the corporate data warehouse that houses laboratory information system data within the National Health Laboratory Service. Weekly Microsoft Excel worksheets were prepared manually and distributed via email prior to the implementation of the interactive dashboard at week 45.

### Percentage of within cut-off turnaround time analysis

**FIGURE 1:**
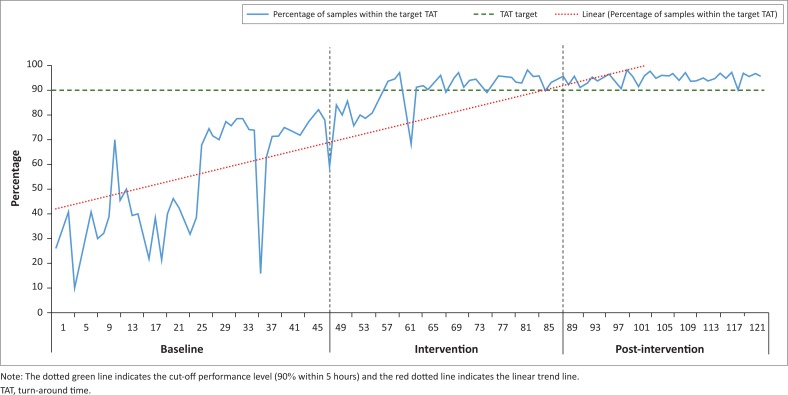
The percentage of within cut-off turn-around times for creatinine testing at a high-volume laboratory across 122 weeks after implementation of a weekly dashboard, Gauteng, South Africa, 2017.

For the baseline phase, the percentage of TAT within the cut-off fluctuated (range: 10% to 79%) (Figure 1). During the intervention, the TAT range again fluctuated from 59% to 97%. For the post-intervention phase, the percentage of TAT with the cut-off ranged from 89% to 98%. The 90% cut-off was met for 42 consecutive weeks from week 81 to the end of the study period.

### 75th percentile turnaround time analysis

**FIGURE 2:**
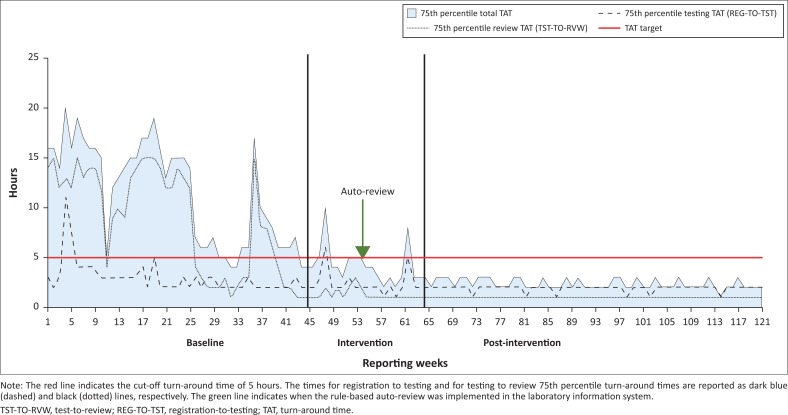
75th percentile total turn-around time for creatinine testing at a high-volume laboratory across 122 weeks after implementation of an interactive weekly dashboard, Gauteng, South Africa, 2017.

During the baseline phase, the total TAT 75th percentile ranged from 4 h to 20 h, changing to 2–10 h for the intervention phase (Figure 2). For the post-intervention phase, the 75th percentile range was 2–3 h. For testing TAT, the 75th percentile for the baseline phase ranged from 2 h to 11 h and changed during the intervention phase to 1–6 h. In the post-intervention phase, the range was 1–2 h. In the baseline phase, the 75th percentile review TAT ranged from 1 h to 15 h compared to 1–3 h for the intervention phase. The post-intervention phase reported a 75th percentile review TAT of 1 h or less.

### Root cause analysis

**FIGURE 3:**
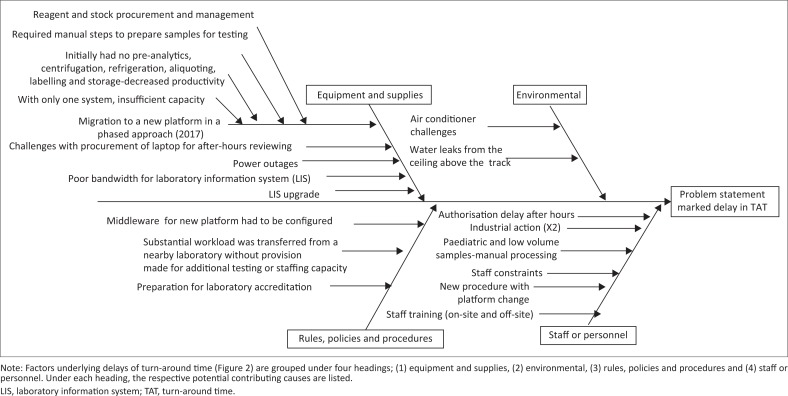
Root cause analysis diagram developed in conjunction with the laboratory manager at a high-volume laboratory, Gauteng, South Africa, 2017.

Four major clusters of contributing causes were identified in the root cause analysis including: equipment and supplies, environmental causes, rules, policies and procedural causes and, lastly, staff and personnel factors (Figure 3). With respect to equipment and supplies, the following causes were shown to have negatively impacted TAT: (1) migration to the new platform (phased approach), (2) difficulties with procurement of laptops to facilitate after-hours off-site authorisation, (3) power outages, (4) bandwidth challenges (laboratory information system [LIS]), (5) LIS upgrade and (6) reagent or stock procurement. For rules, policies and procedures, the following problems were identified: (1) middleware had to be configured, tested and amended due to the changes brought about by a phased approach and (2) a substantial workload was transferred from a nearby laboratory without provision made for additional testing or staffing capacity. Insufficient air conditioning and water leaks from the ceiling were highlighted as causative environmental factors. For staff and personnel considerations, the following were identified: (1) after-hours authorisation delay (by pathologists), (2) industrial action leading to delays, (3) paediatric and low-volume samples requiring manual processing caused bottlenecks in the workflow, (4) staff constraints (in terms of insufficient staff to manage the benches), (5) additional training of staff was required for new procedures and processes for the platform testing changes implemented, (6) prior to full automation, first-line manual sample preparation was needed to enable sample testing and, finally, (7) training for the new platforms provided occurred on site, but staff were also required to attend training off site leaving benches poorly staffed during training periods.

## Discussion

In this study, it was hypothesised that the application of appropriate corrective action guided by an interactive TAT dashboard indicating the proportion of samples within stipulated TAT cut-offs and tail size (outliers) would result in improved performance.^1,10^ This was based on the assumption that delivering an interactive TAT dashboard indicating outlier performance on its own would not result in improvement. Good laboratory management and response with appropriate corrective action is the key catalyst to deliver a sustained quality of reporting and ensure the continual TAT improvement of performance over time.^11^

All laboratories typically adhere to a quality management system (QMS) that is used to assess laboratory quality from the pre-analytic phase through the testing and reviewing processes. A QMS is defined as a set of coordinated activities that direct and control a laboratory with regard to quality.^12^ All aspects of the laboratory operation, including the organisational structure, processes and procedures, need to be addressed by the QMS to assure quality.^12^ Laboratory quality can be defined in terms of accuracy, reliability and timeliness (i.e. TAT).^12^ One of the key practices for continuous improvement is the management review meeting, allowing the laboratory an opportunity to review annual performance as set out in the QMS. The management review cycle involves planning, implementing, checking and acting on a quarterly basis to address shortfalls identified to effect continual improvement.^12^

In this study, we reveal how the introduction of a TAT dashboard enabled senior management of the laboratory in question to assess TAT performance for a particular battery of tests that had not met the stipulated TAT cut-off (greater than 65% of results were outside of the stipulated TAT cut-off). Upon introduction of the dashboard, several areas of concern were immediately identified including pre-analytical, analytical and post-analytical factors. With respect to component TAT, assessing specifically the timeframes of registration to testing and testing to release of the report, TAT delays were attributable to delays of review during weeks 1 to 41, with further delays caused by testing (instrument) interruptions during weeks 4 and 48. The root cause analysis revealed several contributing factors categorised as equipment and supplies, rules, policies and procedures, environmental and personnel or staffing issues. Specifically, the introduction of an auto-review rule process played an important role in improving TAT cut-off. A similarly placed high-volume core laboratory in Canada also reported that the implementation of a series of lean approaches in their busy laboratory, including automation and auto-review rules, were effective to more efficiently manage substantial volumes of samples while meeting TAT cut-offs.^13^

Several important lessons learned and documented by the study laboratory could serve as a template for outreach training to help other public sector laboratories achieve similar TAT performance improvements and establish the practices adopted at this site. Key learning outcomes emerged: Firstly, the importance of the need to collate and actively review real-time information about TAT, including components of TAT, in ensuring overall timely reporting in laboratories was understood and confirmed. Secondly, the value of vertical audits was demonstrated. Vertical audits assisted in understanding what contributed to delayed TAT, and specific focus on outlier samples and vertical audits directed subsequent meaningful corrective action. In line with the requirement of ongoing improvement of service delivery, a weekly ‘results for action’ statement was developed and found to be useful to deliver specific information at the sample or episode level. Such reports, while getting the attention of senior management, could be directed to relevant managers to highlight specific problematic areas and guide the focus of managers’ attention to the investigation of true TAT outliers or exceptions. Such investigation (with solutions) of specifically identified problem areas could yield practical and advantageous outcomes, not only solving the issues at hand but more widely having a positive impact on overall service delivery improvement. The final lesson learned revolved around the importance of documenting and following through on corrective actions as part of the QMS. This ensures that corrective actions taken have consequences and are sustained. In the services review presented here, the week-by-week reminders of outlying TAT were a constant cue that solutions implemented had not been effective. Re-evaluation and re-assessment allowed for streamlined processes to be considered when initial corrective actions had failed. Also highlighted was the importance of conducting a root cause analysis, as cause and effect diagrams, to tease out and understand all aspects of errors and any contributing factors that may lead to delays in TAT. It is also important to point out that although a corrective action may be resolved with a single intervention, more frequently corrective action is a multi-step process to identify possible solutions and alternatives. Once implemented, these corrective steps require consistent monitoring and evaluation for sustained impact. Here, the information provided by the dashboard offered objective evidence of identified issues that could be documented and presented to senior managers month to month and at the annual management meeting; that, in due course, enabled corrective action planning and the facilitated, necessary, mandates to effect better service.

### Past approaches to improving turnaround times

Over the years there have been multiple approaches to monitoring TAT reporting with the aim of improving TAT and, in turn, patient care. Approaches range from identifying outliers to implementing Lean Sigma Six to process mapping.^1,14,15,16,17^ One of the earliest approaches described was the identification of TAT outliers.^1^ Holland et al. reported that the average length of stay in the emergency department across 11 hospitals correlated significantly with the percentage of outliers.^15^ In 2007, Hawkins defined outliers as the TAT of samples that exceeded the institution’s agreed TAT cut-offs.^1^ Outliers could also be defined as the tail size given the skewed TAT distribution. Therefore, the mean and confidence interval are not appropriate measures for assessing TAT performance. In 2018, Coetzee et al. used the 75th percentile and the percentage of samples within the cut-off TAT to identify outliers at the laboratory and test levels.^10^ The development of routine monitoring systems to identify laboratories with a long tail size enabled focused interventions to proactively resolve poor service delivery.^10^

Many laboratories have implemented Lean Six Sigma approaches to improve TAT performance.^13^ Lean is defined as a continuous improvement system consisting of technical tools and management methods.^16^ One of the aims of a ‘lean’ approach is to find and eliminate waste.^16^ For example, waste may be introduced by the layout of a laboratory or by poor process design.^16^ Long distances between the receiving office and testing laboratory could also, for example, encourage staff to move samples in batches resulting in TAT delays.^16^ Padgett et al. reported that the introduction of lean approaches for troponin testing resulted in substantial TAT improvements and averted a proposed point-of-care testing implementation.^16^ Stapleton et al. reported how a lean approach implemented over a three-year period in the laboratory resulted in TAT reductions for all emergency tests^17^ by implementing both workflow improvements and a dedicated emergency bench.^17^

Another approach to improve TAT reported by Barakauskas et al. involved using LIS time stamps, direct observations and discussions with staff to construct various value stream and process maps for immunosuppressant drug level testing.^14^ The value stream map identified process bottlenecks that were addressed.^14^ The process map was reported in columns to represent the major groups of personnel and locations from the health care worker to the reference testing laboratory^14^ with the sequence of events and steps involved illustrated in a vertical direction.^14^ Bottlenecks were also identified in the process map to plan improvement initiatives, for example emergency bag usage.^14^

Ultimately, the aim of any of the approaches described above is to improve TAT performance and, thus, patient care. Although TAT is especially important for emergency tests that have very short TAT cut-offs, it is equally important to set cut-offs for other, less urgent tests to ensure that respective test results are received by attending physicians in a timely fashion to effect appropriate patient care, an important factor in assuring both the quality of care and the cost-effective use of hospital services.^18^ Aside from patient care, TAT delays also have the potential to waste valuable health resources caused by duplicate test requests, thereby increasing public health expenditure.^18^ In summary, the multiple approaches to improving TAT performance across all laboratory tests play an important role in improving the quality of patient care.

### Application to African contexts

**FIGURE 4:**
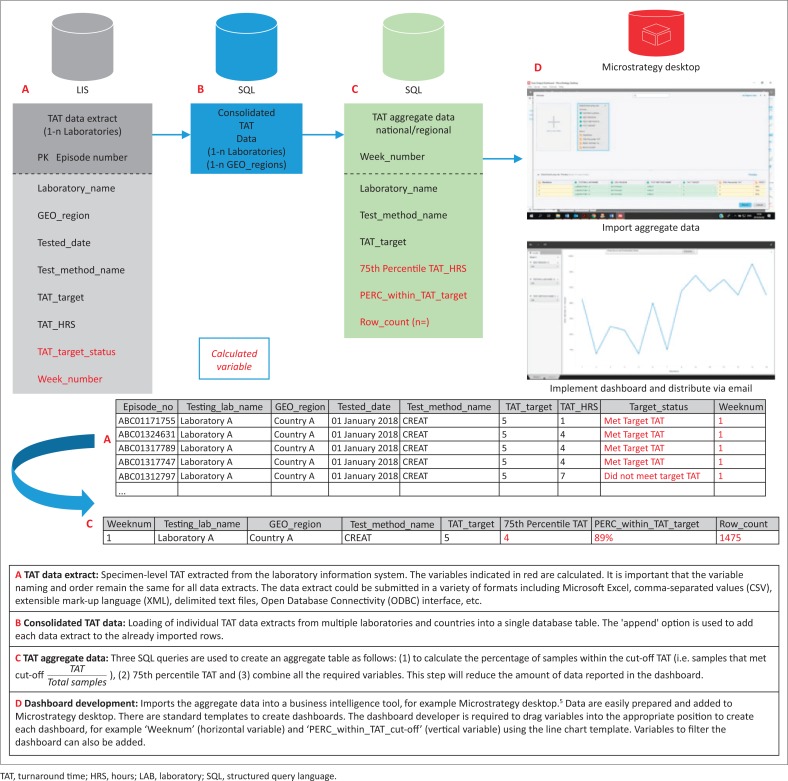
Integration of laboratory information system specimen-level turnaround time data from multiple laboratories into a single, aggregated structured query language database for development of an interactive dashboard, Gauteng, South Africa, 2017.

Figure 4 describes an approach that would make it possible for laboratories in low- and middle-income countries to collate the data required to develop the TAT reporting described in our study. In the first instance, laboratories would require a basic LIS that could generate weekly data extracts as described in Figure 4, using open source database software such as Microsoft SQL Server (Express, Redmond, California, United States).^19^ Equally, ‘MySQL’,^20^ ‘Firebird’^21^ or ‘Cubrid’^22^ could be deployed to generate the aggregate data described. Training is freely accessible via the Internet for these software packages. There are multiple free online courses by providers such as ‘edX’, for example, where one can learn how to both develop and query a structured query language (SQL) database.^23,24^ Any of these software packages could be implemented on a local desktop or in a server environment, depending on the data volume, with very basic query tools using SQL commands making it possible to develop dashboard tools using the step-by-step building blocks approach described in Figure 4.

Cross country collaboration and sharing of resources could play an important role in securing already developed dashboard tools for other African countries. A multi-country approach could reduce overall costs and effort. For example, a single TAT dashboard could be developed for the Southern African Development Community to ensure accessibility and provide scalability. To secure the system and provide confidentiality, each country could have access to their own data using data access privileges. The benefit of this approach is that after the methods, systems and dashboards are developed, it is easy to extend these developments to other countries with minimal additional cost. The only additional effort required at the country level would be to collate and share the data extracts with the umbrella organisers.

### Limitations

Only LIS data were used for our study. Without a laboratory specimen tracking system, it is not possible to report end-to-end TAT. The implementation of an end-to-end tracking system from the time of venesection to delivery into a laboratory, additionally integrated into a TAT dashboard, could provide valuable supplementary date and time values to allow for an extended TAT efficiency review.

### Conclusion

In summary, this study demonstrated that an interactive TAT dashboard, reporting appropriate TAT parameters, applied in the context of a QMS, coupled with proactive and diligent management, can accurately identify outliers and lead to appropriate corrective action and sustained timely laboratory reporting.

Lessons learned

- A weekly interactive TAT dashboard enables reporting of appropriate TAT parameters and respective outcomes by confirming ongoing quality and timely reporting, as well as identifying outlying TAT that may require appropriate corrective action.
- TAT data can be collected at the laboratory, local network or national level. A dashboard that includes aggregated and local level data, with a data drilling function, allows hierarchical review of the data, so that both higher-level managers and laboratory managers are able to view the same data, but at different levels appropriate for their respective level of responsibility.
- Continuously collating and analysing the data and presenting TAT information in a user-friendly, visual dashboard format allows for immediate attention to be focused on outlying sites and areas.
- Visibility and transparency of TAT data and outcomes to all levels of management provides an incentive (with repeated peer or organisation pressure, if consistently outside of TAT) to act on poor performance.
- A quality management system requires active input, monitoring and appropriate action where needed. The presentation of information does not necessarily confer good performance or the meeting of TAT cut-offs. A dashboard, such as that presented here, is merely a tool. Proactive, consistent and diligent review of TAT data presented in a dashboard is required to facilitate meaningful improvement and corrective action. An auto-review rule implemented for a specific test or battery of tests on the laboratory information system has the potential to reduce TAT by acting to reduce the workload for senior staff through automatic review of predominantly normal results. With the auto-review implemented, senior staff effectively use their time and reserve the resultant review only for samples that fail to meet the auto-review rule, for example delta check failures.
